# Comparison of IGS 2 score and a new neural network biomarker score performance in predicting 1-year mortality

**DOI:** 10.1186/cc12418

**Published:** 2013-03-19

**Authors:** C Romdhani, I Labbene, M Belhadj Amor, Z Hajjej, A Massoudi, M Ferjani

**Affiliations:** 1 Military Hospital of Tunis, Tunisia

## Introduction

The aim of this study was to compare the IGS 2 score with a predictive model built using clinical characteristics and biomarkers associated with 1-year mortality from procalcitonin, C-reactive protein, troponin I (TnI) and NT-Pro-BNP.

## Methods

We conducted a prospective observational cohort study in a polyvalent ICU. We calculated the IGS 2 severity score in the first 24 hours. We dosed NT-Pro-BNP, TnI, procalcitonin and C-reactive protein at admission to the ICU. To test the biomarker model, the patient database was divided into training (70%) and test (30%) databases. A neural network model was trained from variables associated with 1-year mortality. All statistics were done using R v.2.15.1. We used the pROC package to plot and compare ROC curves.

## Results

**Figure 1:**
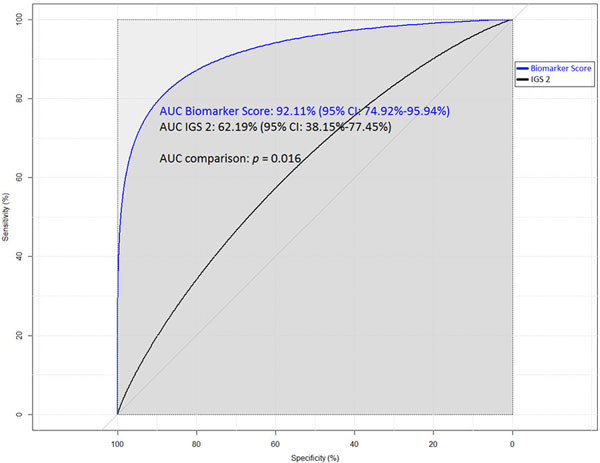
**Comparison of area under ROC curves (AUC) of the biomarker score and IGS 2**.

Lower age, NT-Pro-BNP and TnI were significantly associated with 1-year survival. We built a neural network model using the training database. We used the test database to compare our model with the IGS 2 score. The difference between the AUC of the biomarker score and the IGS 2 score was statistically significant (Figure 1).

## Conclusion

Our new neural network model built using age, NT-PRO-BNP and TnI succeeded the internal-validation processes and predicts 1-year mortality better than IGS 2.

